# A Review of Selected IBD Biomarkers: From Animal Models to Bedside

**DOI:** 10.3390/diagnostics11020207

**Published:** 2021-01-30

**Authors:** Emiko Mizoguchi, Renuka Subramaniam, Toshiyuki Okada, Atsushi Mizoguchi

**Affiliations:** 1 Department of Immunology, Kurume University School of Medicine, 67 Asahi-machi, Fukuoka 830-0011, Japan; okada_toshiyuki@med.kurume-u.ac.jp (T.O.); mizoguchi_atsushi@med.kurume-u.ac.jp (A.M.); 2 Department of Molecular Microbiology and Immunology, Brown University Alpert Medical School, Providence, RI 02912, USA; 3 Department of Surgery, Brigham and Women’s Hospital and Harvard Medical School, Boston, MA 02115, USA; rsubramaniam1@bwh.harvard.edu

**Keywords:** inflammatory bowel disease, biomarker, colitis-associated cancer, ANCA, chitinase 3-like 1

## Abstract

Inflammatory bowel disease (IBD) is a dysregulated inflammatory condition induced by multiple factors. The etiology of IBD is largely unknown, and the disease progression and prognosis are variable and unpredictable with uncontrolled disease behavior. Monitoring the status of chronic colitis closely is challenging for physicians, because the assessment of disease activity and severity require invasive methods. Using laboratory biomarkers may provide a useful alternative to invasive methods in the diagnosis and management of IBD. Furthermore, patients with ulcerative colitis or Crohn’s disease are also at risk of developing cancer. Annual colonoscopies can help lower the risk for developing colorectal cancer. However, laboratory biomarkers may also be helpful as non-invasive indicators in predicting treatment responses, improving prognosis, and predicting possible tumors. This review addresses selected laboratory biomarkers (including ANCA, chitinase 3-like 1, S100A12/RAGE, calprotectin, and TNF/TNFR2), which are identified by utilizing two well-accepted animal models of colitis, dextran sodium sulfate-induced and T cell receptor alpha knockout colitis models. In addition to being useful for monitoring disease severity, these biomarkers are associated with therapeutic strategies. The factors may regulate the initiation and perpetuation of inflammatory factors in the gut.

## 1. Introduction

Inflammatory bowel disease (IBD), including ulcerative colitis (UC) and Crohn’s disease (CD), is a group of intestinal inflammatory conditions that affects more than two million people in the United States. It has been gradually revealed that the etiology and pathogenesis of IBD are more complicated than initially imagined, due to the existence of perturbational complexity in specific microbial strains, transcriptional changes, and metabolic alterations, which are closely associated during the development of this disease [1]. A genome-wide association study (GWAS) conducted by multiple worldwide institutions found that over 200 regions of the human genome are associated with IBD susceptibility [2,3]. Based on the results, the IL-10 receptor and its signaling components seem to be critical in regulating homeostasis and immunity in the intestine. Polymorphisms in genes encoding for IL-10RA and/or IL-10RB are closely associated with human IBD and can lead to the onset of disease as early as three months of age [4,5]. 

Animal models are genuinely beneficial in studying the mechanistic details during the development of IBD. The T cell receptor alpha chain (TCRα) knockout (KO) animal model, established in 1993, is one of the three major genetically manipulated murine models of spontaneous colitis [6]. The inflammation in TCRαKO mice is similar to UC because it is restricted to the colonic mucosa with the pathological findings of goblet cell depletion, crypt elongation, and cellular infiltration into the lamina propria [6]. Unique CD4^+^ TCRα^−^ β^+^ T cells in colonic lamina propria actively produce IL-4, one of the major Th2 types of cytokines, which results in spontaneous colitis development [7]. Of interest, similar to UC, approximately 70% of TCRαKO mice produce anti-neutrophil cytoplasmic antibodies (ANCA), most of which are perinuclear staining pattern (p-ANCA), in the diseased colonic mucosa [8]. Interestingly and surprisingly, B cells, immunoglobulins, and autoantibodies in TCRαKO mice are not necessary to induce spontaneous chronic colitis, but have a suppressive role [9]. It has been found that the regulatory type of B cells (also known as Breg) existing in TCRαKO mice produce large amounts of IL-10 or IL-12p70 and contribute to suppressing the development of chronic colitis [10]. From these results, autoantibodies including ANCA may not be associated with a role in the pathogenesis of colonic inflammation. Based on our experimental results on IBD, the TCRαKO mouse model seems to be one of the best spontaneous chronic colitis models because it closely reflects the characteristics of human UC [11]. 

The dextran sodium sulfate (DSS)-induced colitis model is another frequently utilized animal model, originally reported by Okayasu et al. in 1990 [12]. In this model, extensive colonic epithelial injury with complete basal crypt depletion has been observed after one cycle of 2.5–5% DSS administration for 4–7 days, followed by regular water replacement. A few days after the cessation of DSS, the injured colonic tissue starts showing relatively slow regeneration with extensive crypt and epithelial damage, mononuclear cell infiltration, tissue edema, and scattered severe ulceration [13]. Acquired immune responses are not related to the induction of DSS-induced colitis because severe combined immunodeficiency (SCID) also causes severe mucosal damage by DSS administration [14]. In addition, chronic colitis can be induced with 3–5 cycles of DSS: each cycle consists of 4–7 days administration of DSS followed by regular water for another 7–10 days [12]. This long-term DSS administration induces low-grade colorectal dysplasia, high-grade dysplasia, and adenocarcinoma, which is relatively similar to the course of tumor development (dysplasia-carcinoma sequence) in UC [13]. DSS-induced colitis is very reproducible because it does not require any special techniques for the administration of DSS. However, environmental factors, especially cleanliness of the animal facility, are closely associated with the severity of DSS-induced colitis: under sterile conditions or by treating the mice with mixed antibiotics, more severe colitis has been observed with increased production of proinflammatory cytokines including IL-1β, TNFα and IL-6 [13]. This observation suggests the importance of balance in commensal bacteria during the development of DSS-induced colitis.

This review article has summarized a few selected biomarkers, which were identified by utilizing the above two animal models of IBD. In the following sections, we have discussed that these biomarkers can be exploited to develop future diagnostic strategies for IBD, including UC, CD, and indistinguishable colitis. Selected biomarkers for colitis-associated cancer have also been discussed. 

## 2. A Few Selected IBD Biomarkers, Which Are Identified by Murine Experimental Models of DSS-Induced Colitis and/or TCRα KO Mice

### 2.1. pANCA

ANCAs react with antigens in the cytoplasm of neutrophils. Two distinct patterns of staining have been reported: cytoplasmic pattern (cANCAs) and perinuclear pattern (pANCAs). ANCAs were reported in UC patients for the first time in 1990 [15]. pANCAs are produced in response to intestinal bacterial antigen. UC-pANCA reactive antigen has been identified as a PKKAK motif of histone H1 [16,17]. Meanwhile, several other reports claim that reactive antigens for UC-pANCA have not yet been elucidated. Mizoguchi et al. showed that 70% of the sera from the chronic colitis animal model, TCRα KO mice, reacted with human neutrophil extract and exhibited pANCA pattern [8]. Unlike in vasculitis, pANCAs from UC patients did not cause respiratory burst in normal human neutrophils in vitro, which suggests that pANCAs are unlikely to play role in UC pathogenesis [18].

At present, several human studies have showed pANCAs in sera of approximately 60–70% of UC, 10–15% of CD patients and <5% in non-IBD colitis patients [19,20]. The presence of pANCAs is relatively consistent over time in UC patients, but concentrations change with treatments, particularly immunosuppressive drugs [21]. pANCA levels were significantly higher in treatment-resistant left-sided UC patients compared to treatment-responsive UC patient groups [18]. pANCA levels significantly increase in CD patients with UC clinical symptoms (left side colitis); therefore, its use for IBD sub-classification becomes limited [22]. Studies suggest using ASCA (antibodies for Saccharomyces cerevisiae)/pANCA combination could be used to differentiate UC versus CD. pANCA titers are higher in UC, whereas ASCA titers are higher in CD. However, meta-analysis of 60 studies comprising 3841 UC and 4019 CD patients revealed that pANCA/ASCA tests are specific for UC but not sensitive for CD [17]. pANCA+/ASCA− results for UC showed 51% sensitivity and 94% specificity. pANCA−/ASCA+ results for CD showed 55% sensitivity and 94% specificity. The pediatric subgroup with UC/CD analysis showed 70.3% sensitivity and 93.4% specificity when a pANCA+/ASCA− combine test was performed. Therefore, the pANCA/ASCA combination test may be useful in differentiating UC from CD in pediatric populations. Although the sensitivity of the assays limits their use as screening tools, high specificity of these assays could be used for diagnostics.

Furthermore, pANCA has been shown to be more sensitive in Caucasian than Chinese UC patients [23]. UC–pANCAs activity is limited to neutrophils, and titers do not correlate with the severity or the duration of the disease. Studies have shown a strong correlation between proteinase-3 ANCAs (PR3-ANCA) in the serum and UC disease courses [24,25]. These findings suggest that ANCAs could play a role as a biomarker for diagnosis, as well as screening, prediction of disease status, or responsiveness to treatment. However, further research is required to validate ANCA use in adult and pediatric IBD populations.

### 2.2. Chitinase 3-Like 1 (CHI3L1)

CHI3-L1 is a secreted glycoprotein, which belongs to the glycoside hydrolase 18 family of chitinases. A chitin binding domain at the C-terminus is enzymatically inactive. It is secreted by various cell types, including intestinal epithelial cells/colonocytes, neutrophils, fibroblast-like synovial cells, vascular smooth muscle cells, hepatic stellate cells, and monocytes/macrophages. CHI3L1 binds with RAGE (receptor for advance glycation end product), IL-13Rα2 and Syndecan-1/αVβ3 integrin [26]. CHI3L1 is not expressed or secreted under healthy conditions, but rather specifically under inflammatory conditions. CHI3L1 regulates multiple cellular responses, including inflammation, cell proliferation, apoptosis, pyroptosis, inflammasome activation, wound healing, tumorigenesis, and angiogenesis, due to its ability to bind with different receptors. In addition, CHI3L1 enhances bacterial invasion as well as adhesion to epithelial cells [27,28]. Furthermore, our group has demonstrated that adherent invasive *Escherichia coli* (AIEC) adheres to N-glycosylated CHI3L1 in intestinal epithelial cells and exerts its pathogenic effect in an AIEC-infectious colitis model [28].

CHI3L1 has been detected in colonic epithelial cells, stools, and sera of patients with IBD and has been suggested as a potential biomarker. Previous studies by Mizoguchi et al. reported CHI3L1 expression in colonic epithelial cells and lamina propria macrophages in DSS-induced colitis and IBD patients [27,28]. Chen and Mizoguchi et al. showed significantly increased mRNA expression (by real-time PCR) of CHI3L1 in the colonic epithelial cells of patients, with UC and UC with remote dysplasia as compared to healthy individuals [29]. CHI3L1 expression was significantly increased in patients with UC who had remote dysplasia, while there was a less significant increase in UC patients without dysplasia compared to healthy individuals [29]. Following real-time PCR analysis, it was revealed that CHI3L1 expression had an approximately 20-fold increase in patients with UC with remote dysplasia as compared to healthy individuals. This increase suggests that CHI3L1 in colonic biopsy specimens in patients with chronic UC seems to be strongly associated with neoplastic change/transformation of the colonic epithelium [29].

Chronic inflammation in the large intestine is one of the major risk factors for colitis-associated cancer (CAC). Accumulated evidence supports the fact that soluble factors secreted by epithelial cells promote CAC by preventing cancer cell apoptosis, modulating inflammation, and stimulating angiogenesis [26,29]. Colonic CHI3L1 mRNA expression also increased in active UC and CD patients but within the normal range in UC remission and unaffected areas of CD [27]. Our studies using WT and CHI3L1 KO with and without a bone marrow transplant also reiterate the important role of non-hematopoietic cell-derived CHI3L1 in the development of CAC in an azoxymethane/DSS-treated mouse model [30].

Feces is a reliable source to measure mucosal inflammation directly in a non-invasive manner. Increased levels of fecal CHI3L1 have been reported in pediatric as well as adult IBD patients, as summarized in Table 1. Aomatsu et al. used 13.7 ng/g fecal CHI3L1 as a cutoff to differentiate a healthy control from IBD (UC and CD) pediatric patients. Median CHI3L1 levels of active UC and CD patients were 366.6 ng/g (*n* = 44) and 632.7 ng/g (*n* = 48), respectively [31]. The CHI3L1 levels strongly correlated not only with clinical/endoscopic disease severity of both UC and CD patient cohorts, but also with calprotectin levels. Fecal CHI3L1 levels also decreased post treatment remission. Fecal CHI3L1 levels more than 15 ng/g were able to predict endoscopic activity in UC patients with 81.8% sensitivity and 80% specificity. Of note, fecal biomarkers are very useful for detecting remission, early relapses, and efficacy of anti-inflammatory treatment in IBD patients. Our group analyzed fecal CHI3L1 levels in fecal samples of healthy, IBD remission, adenoma, sporadic adenocarcinoma, and IBD–CAC patient cohorts. Significantly increased levels of CHI3L1 were observed in the IBD–CAC cohort compared to the rest of the groups [30]. Further studies will be required in adult and pediatric IBD patients to completely validate fecal CHI3L1 as a reliable diagnostic biomarker during the neoplastic changes of colonic epithelium.

In a human study, serum CHI3L1 levels were measured in patient cohorts of UC, CD, and matched healthy controls, as well as intestinal inflammation of other causes. The mean serum levels were significantly higher in UC (102.6 ± 82.7 ng/mL) and in CD (112.2 ± 83.7 ng/mL). Healthy controls (64.1 ± 21.4 ng/mL) and other intestinal inflammatory controls (77.8 ± 23.1 ng/mL) had comparable levels of CHI3L1 in sera [32]. The quantity of CHI3L1 was positively associated with the degree of inflammation and disease activity, but not with disease type. Serum CHI3L1 concentrations were significantly higher in CD patients with a stricture formation (167.5 ± 119.30 ng/mL) than that of healthy controls (44.92 ± 24.89 ng/mL) or CD patients without stricture formation (80.12 ± 56.38 ng/mL) [33]. A study by Vind et al. concluded that 40–50% of UC and CD patients with active disease have higher levels of CHI3L1 [34]. There was low correlation between levels of CHI3L1 and CRP. Increased CHI3L1 levels also positively associated with poor prognosis of IBD [30,34].

In summary, a unique association of epithelial, fecal, and serum CHI3L1 levels with IBD has been shown in various human studies. However, sensitivity and specificity of these assays should be evaluated carefully. Further human studies are warranted to implement CHI3L1 as a potential non-invasive diagnostic biomarker for IBD and CAC.

### 2.3. S100A12/RAGE

The granulocytic protein S100 calcium-binding protein A12 (S100A12), also known as EN-RAGE (extracellular newly identified RAGE-binding protein), Calgranulin C, and MRP6 (Myeloid-related protein 6), belongs to the S100/Calgranulin member of the EF-hand family of calcium-binding proteins and is mainly expressed in neutrophils, monocytes, and macrophages. It is also found in endothelial cells, keratinocytes, and epithelial cells under inflammatory conditions [35]. S100A12 is known to be a ligand for RAGE [36], toll-like receptor 4 (TLR4) [37,38], and CD36 (also known as platelet glycoprotein 4) [39,40], which are receptors for advanced glycation end products (AGEs) expressed on monocytes and epithelial cells. S100A12 exists in a variety of forms, including homodimers and hexamers [41], and binds to the V- or C-type domains (V and C1 domains) of RAGE, which are well known as pattern recognition receptors (PRRs). Therefore, it is thought to be involved in the activation, survival, and mobilization of RAGE-expressing T cells, B cells, dendritic cells, and macrophages [42]. In addition, the extracellular domain of RAGE is usually known to bind to various ligands such as high-mobility group box 1 (HMGB1) proteins [43], AGEs [44], transthyretin [45], DNA [46], and β-amyloids [47]. The characteristics of S100A12 and RAGE are summarized in Table 2.

In the respiratory system, S100A12 secreted by neutrophils binds to RAGE expressed on airway epithelial cells, which predominantly activates the extracellular signal-regulated kinase (ERK) pathway rather than the nuclear factor kappa beta (NF-kB) pathway and promotes the production of MUC5AC, which is a major component of respiratory mucin protein [48]. In addition, S100A12 has various extracellular activities that contribute to the innate immune response, such as the activation of intracellular signaling cascades to produce cytokines in parallel with the induction of oxidative stress and chemotaxis activity [49]. The intestinal mucosa of patients with active IBD can be characterized by an active infiltration of neutrophils, and S100A12 is reported to be secreted by the activated neutrophils and other granulocytes [50,51]. The expression and secretion of S100A12 are elevated in the serum and tissues of IBD patients [52,53,54]. In addition, fecal S100A12 may be useful for an indicator of mucosal healing in IBD and a predictive marker of severe UC [53,55]. However, fecal S100A12 cannot be used to distinguish UC from CD and shows a weak correlation with clinical and histological severity scores of UC patients. Therefore, S100A12 may not be one of the best markers for predicting or monitoring the responses for IBD treatments. Discrimination between mild IBD and irritable bowel syndrome (IBS) may not be easy, and in this case, fecal S100A12 has been shown to be a useful marker to distinguish between the two diseases with a sensitivity of 86% and specificity of 96% [56]. Despite the fact that S100A12 is uniformly distributed in feces and stable for seven days at room temperature, it is not widely used in clinical practice [57].

### 2.4. S100A8/S100A9: Calprotectin

The L1 protein, first isolated in granulocytes in 1980 [58], was later named calprotectin because it was found to retain calcium-binding and antibacterial properties [59,60]. Calprotectin, composed of a complex of S100A8 (MRP8, Calgranulin A) and S100A9 (MRP14, Calgranulin B), is a Ca^2+^, Zn^2+^-binding protein belonging to the S100 family and is produced by granulocytes, monocytes, macrophages, and epithelial cells under inflammatory conditions in animal models of colitis and human IBD [61,62]. S100A8 and S100A9 are known to account for approximately 60% of cytoplasmic proteins and 5% of total proteins in neutrophils [63,64]. Interestingly, in DSS-induced colitis, the upregulation of S100A8 and S100A9 by colonic epithelial cells was much greater during the recovery phase than in the acute phase of colitis [61]. Low and Mizoguchi et al. also identified that CHI3L1 binds to the RAGE receptor and cuts the S100A9-mediated positive feedback loop, leading to the downregulated expression of S100A9 in the chronic DSS-induced colitis model [30].

Extracellular calprotectin (a heterodimer of S100A8 and S100A9), which is known to act as endogenous damage-associated molecular patterns (DAMPs), is secreted by cell disruption, cell death, and cell activation (e.g., released by stressed cells during intestinal inflammation). The secreted calprotectin binds to pattern recognition receptors (PRRs) including TLR4, RAGE, and EMMPRIN (also known as CD147 or Basigin) [65,66], heparan sulfate proteoglycans, and carboxylated N-glycans on the surface of endothelial cells. It also induces the activation of NF-kB and mitogen-activated protein kinase (MAPK), suggesting that calprotectin is involved in positive feedback of inflammation [62,67]. In addition, experimental results from S100A9-deficient mice have shown that deletion of S100A9 results in the protein instability of S100A8, which in turn leads to the deletion of both S100A8 and S100A9 proteins [62,68,69]. A summary of the characteristics of calprotectin (S100A8/S100A9) is shown in Table 2. Blood samples such as CRP, hemoglobin, albumin, and fecal occult blood have been used to monitor IBD. However, the specificity of CRP in IBD is high (92%), while its sensitivity is low (49%), which has been cited as a problem [60]. Calprotectin in serum and plasma has been found to be elevated in a variety of inflammatory diseases, including IBD, psoriasis, and rheumatoid arthritis [62,65,70,71]. In addition, although fecal calprotectin is evenly distributed and can be measured stably for up to seven days at room temperature [72], it is gradually degraded starting three days after collection (28% decrease by day seven [73,74]. Fecal calprotectin is often used as a non-invasive adjunctive diagnostic marker and relapse risk marker that correlates well with the severity of endoscopic findings in patients with IBD, including UC and CD (sensitivity 88%, specificity 73%) [60,75]. However, measurement of fecal calprotectin is difficult to use as a substitute for endoscopy due to the lack of assessment of responsiveness to disease changes and lack of validated biomarkers of IBD activity [76]. In addition, false-positives for fecal calprotectin have been found in patients with gastrointestinal diseases and those treated with non-steroidal anti-inflammatory drugs or proton pump inhibitors [77]. Moreover, fecal calprotectin levels in patients with ileal CD, who may have large or extensive ulcers, are not markedly elevated. Therefore, the interpretation of fecal calprotectin levels should also be taken into account for the site of disease [78]. One report suggests that a combination of fecal calprotectin levels and quantitative magnetic resonance activity index (MaRIA) may be effective in monitoring mucosal activity in patients with small intestinal CD [79]. Additionally, a home-used lateral flow test to measure fecal calprotectin has been developed for monitoring IBD activity, suggesting that it may be used for the telemonitoring of patients with asymptomatic IBD [80,81]. 

Fecal calprotectin is predominantly high in the neonatal period, when the gastrointestinal mucosal barrier is immature, compared to normal levels in adults; calprotectin levels gradually decrease after one month of age [82]. In addition, calprotectin levels are predominantly lower in premature infants during the first month of life than in growing infants [83]. Hence, it is more necessary to set a different reference value for calprotectin in children than in adults, especially in those younger than four years. Therefore, the validity and reference values of fecal calprotectin in children have not yet been established, due to the much greater variability than in adults [84,85]. Fecal calprotectin is useful in determining the activity of UC, but it is only an adjunct in determining remission. Calprotectin in neonates has been suggested to be related to diseases involving intestinal bacterial components, due to its potential to regulate the development of the intestinal microbiota and immune system [86].

**Table 2:** Characterization of Calprotectin (S100A8/S100A9) and S100A12.

	Calprotectin	S100A12
Expression	granulocyte, monocyte and macrophage [62] epithelial cell under inflammatory conditions [48,61]	neutrophil, monocyte, and macrophage [35,52] endothelial cell, keratinocyte, and epithelial cell under inflammatory conditions [35,48]
Function	modulate intracellular calcium signaling [65] antibacterial infections [71] regulation of the intestinal microbiota and immune system in neonate [86]	promotion of cytokine production [35,65] regulation of leukocyte adhesion and migration [35,65]
Receptors	TLR4 [67] Heparensulfate proteoglycans [62] *N*-glycans [62] RAGE [65] EMMPRIN [66] (only S100A9 binds)	TLR4 [37,38] RAGE [42] CD36 [39,40]
Related diseases	Inflammatory diseases [67] IBD Psoriasis Rheumatoid arthritis Transplantation Infections	IBD IBS Arthritis Vasculitis Periodontitis Kawasaki disease Infections [39,40,56,65]

EMMPRIN, extracellular matrix metalloproteinase inducer; RAGE, receptor for advanced glycosylation and products; TLR4, toll-like receptor 4; IBD, inflammatory bowel disease; IBS, irritable bowel disease.

### 2.5. Proinflammatory Factors Including IL-1β, TNFα and TNFR2

Cytokines are small proteins (~5–20 kDa) produced by immune cells and play an important role in cell signaling. In particular, proinflammatory cytokines including IL-1β, TNFα, and IL-6 play a crucial role in the immune responses during the development and perpetuation of IBD [5].

In 1997, Mizoguchi et al. identified that in vivo neutralization of IL-1α or IL-1β by intraperitoneally injecting the specific monoclonal antibodies suppressed colonic epithelial proliferation and decreased colonic T cell infiltration in the early stage of chronic colitis in TCRαKO mice [87]. After administering both IL-1α and IL-1β monoclonal antibodies, there were approximately 50% fewer CD3^+^ T cells in both lamina propria lymphocytes and intraepithelial lymphocytes than in the control (hamster serum-treated) TCRαKO mice [87]. Based on the results, IL-1 seemed to be responsible for the development of very early stage of chronic colitis (around eight weeks of age) by increasing the initiation of epithelial proliferation and T cell infiltration [87]. A study by Menghini et al. showed that IL-1α plays a central role in the pathogenesis of DSS-based experimental IBD by enhancing dysbiosis [88]. In addition, autosensing and paracrine sensing of IL-1 seem to be key in the control of IL-23-producing monocytes in subgroups of IBD patients [89]. These results strongly support a pivotal role of IL-1 in IBD development. 

Another critical cytokine for IBD development is TNFα, which regulates immune cell survival and function. Lopetuso et al. reported that locally administered anti-TNFα chimeric antibody (infliximab) significantly ameliorated the severity of DSS-induced colitis on C57Bl/6 mice [90]. Interestingly, in this model, healthy mice showed higher serum and intestinal mucosal, but not fecal infliximab levels, as compared to colitic mice [90]. Similarly, anti-TNF inhibitors, in particular infliximab and adalimumab, have dramatically improved the quality of life (QOL) of IBD patients over the last 15 years [91,92,93]. TNFα induces multiple effects, including altered cell proliferation and cell death through distinct cell signaling cascades resulting from binding to TNF receptor-type I (TNFR1) and -type II (TNFR2) [94]. Anti-TNF treatment successfully inhibits the expression of A20/TNFAIP3 (TNFα-induced protein 3) in memory CD4^+^ T cells mediated through TNFR2 signaling cascade [95]. Of interest, increased expression of inducible TNFR2 was observed on colonic epithelial cells in colitis of TCRαKO mice (around 12 weeks old), as well as patients with UC and CD. Upregulation of TNFR2 on colonic epithelial cells, regulated by IL-6 in the presence of TNFα, seems to be associated with the perpetuation of inflammatory process and altered intestinal epithelial cell functions [96]. These findings suggest that expression of TNFR2 on colonic epithelial cells plays a pivotal role in the colonic inflammation-associated alteration of intestinal epithelium, and therefore intestinal TNFR2 expression will be a good biomarker of IBD severity [96,97]. TNFα can also bind to TNFR1, of which ligation induces the growth arrest through MAPK (mitogen-activated protein kinase) activity [94]. Based on previous observations, TNFR1-mediated innate immune responses significantly enhance host defense against non-pathogenic microorganisms in commensal microflora [98]. Therefore, colonic epithelial TNFR2 but not TNFR1 may be positively associated with the disease severity of IBD [98]. It has been reported by Spoettl et al. that serum levels of soluble TNFR2 are significantly more increased in active CD patients than in UC patients, and could be used to discriminate both diseases [99]. 

## 3. Conclusions

Utilizing the DSS-induced epithelial injury model, as well as the TCRα spontaneous chronic colitis model, several colitis-associated biomarkers have been identified. These markers are very useful and reliable for monitoring the severity of colitis and are significantly associated with the progression of intestinal inflammation and may be helpful in colitis prognosis. It is apparent that no single biomarker is perfect for the diagnosis of IBD, and therefore it is necessary to utilize appropriately mixed biomarkers to define the disease activity, severity, spreading range and prognosis. In particular, CHI3L1 is likely to be a reliable biomarker for colitis-associated epithelial neoplastic changes [30,100] during monitoring IBD patients in the remission phase.

## Figures and Tables

**Table 1:** Use of fecal and serum CHI3L1 as a biomarker and its association with inflammatory bowel disease.

References	Age	Disease Type and Number of Enrolled Patients	Chitinase 3-like 1 Levels	
**Feces**				
Aomatsu et al. (2011) [31]	P	UC (*n* = 94) CD (*n* = 87) HC (*n* = 56)	Active UC	366.6 ng/g (median)
			Active CD	632.7 ng/g
			Inactive UC	15.8 ng/g
			Inactive CD	18.4 ng/g
			HC	2.2 ng/g
**Serum**				
Koutroubakis et al. (2002) [32]	A	UC (*n* = 94)	UC	102.6 ± 82.7 ng/mL
		CD (*n* = 85)	CD	112.2 ± 83.7 ng/mL
		HC (*n* = 70)	HC	64.1 ± 21.4 ng/mL
		Non-IBD (*n* = 23)	Non-IBD	77.8 ± 23.1 ng/mL
Erzin et al. (2007) [33]	A	CD (*n* = 41) HC (*n* = 46)	CD	105.69 ± 88.08 ng/mL
			CD with stricture	167.60 ± 119.3 ng/mL
			CD w/o stricture	80.12 ± 56.38 ng/mL
			HC	44.92 ± 24.89 ng/mL

P, pediatric; A, adult; UC, ulcerative colitis; CD, Crohn’s disease: w/o, without; HC, healthy control.
